# Supplementary material for: Study on the Preparation Method of Quality-Assured In-Hospital Drug Formulation for Children—A Multi-Institutional Collaborative Study
Source: Children (Basel). 2023 Jul 10;10(7):1190. doi: 10.3390/children10071190 (PMC10378225; doi:10.3390/children10071190)
Supplement: Supplementary file 1 [file children-10-01190-s001.zip › children-2470723-supplementary.pdf]

Supplementary materials

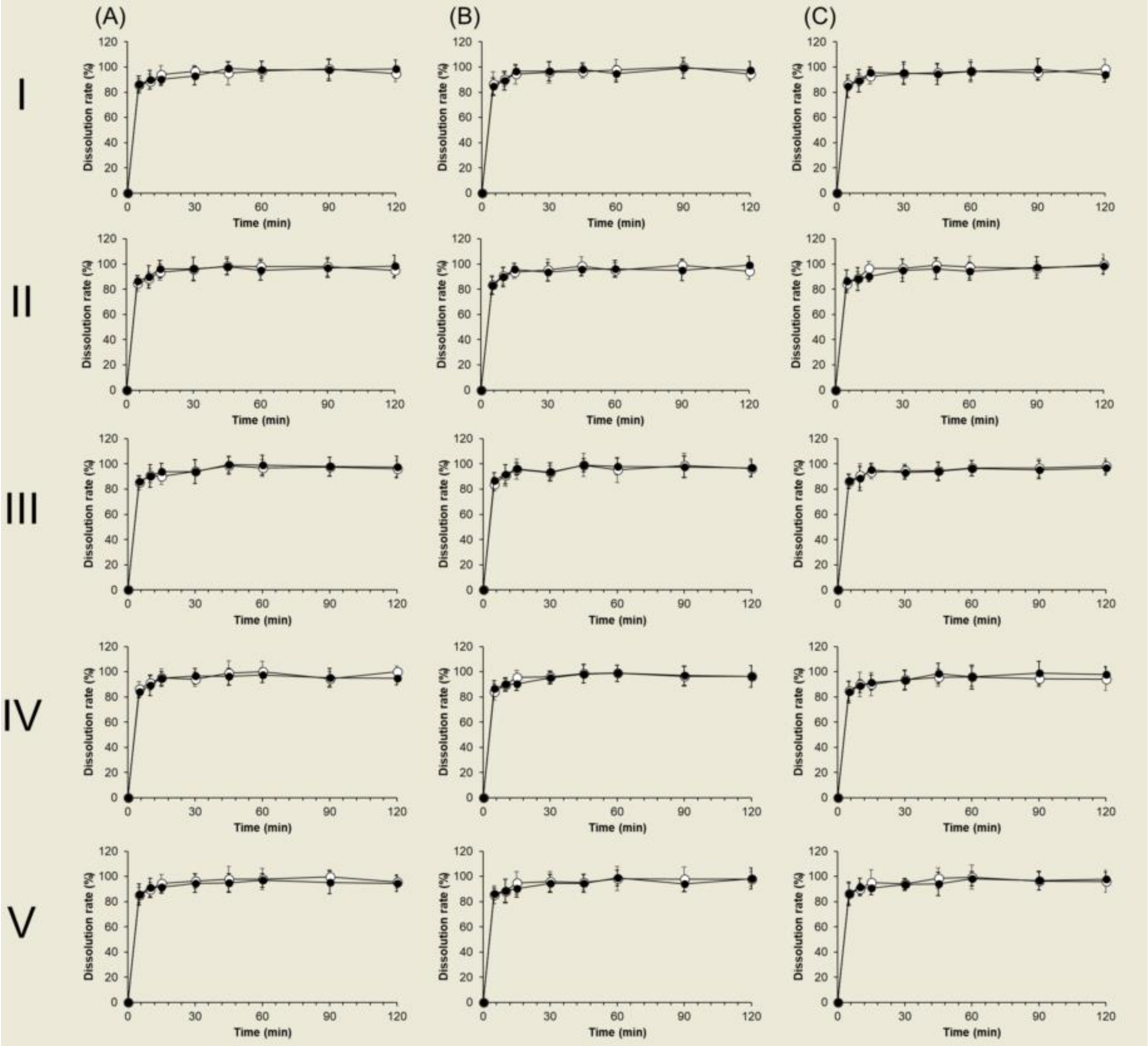

Figure S1. Dissolution profiles of compounded baclofen powder in water stored in three conditions: closed bottle (A), in-use bottle (B), and laminated paper (C). Open circles indicate the dissolution rate on day 0; closed circles indicate the dissolution rate on day 120.

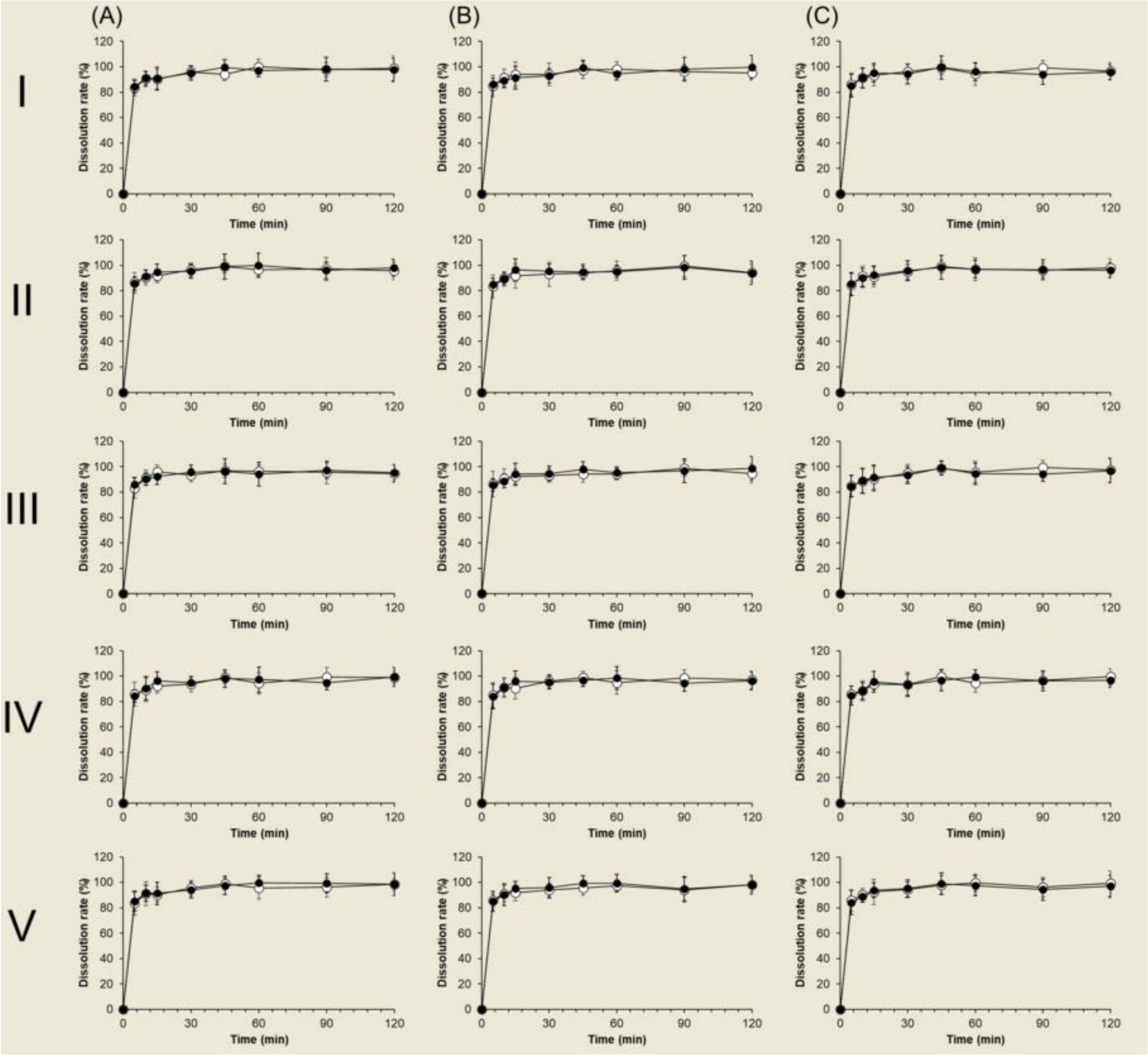

Figure S2. Dissolution profiles of compounded clonidine powder in water stored in three conditions: closed bottle (A), in-use bottle (B), and laminated paper (C). Open circles indicate the dissolution rate on day 0; closed circles indicate the dissolution rate on day 120.

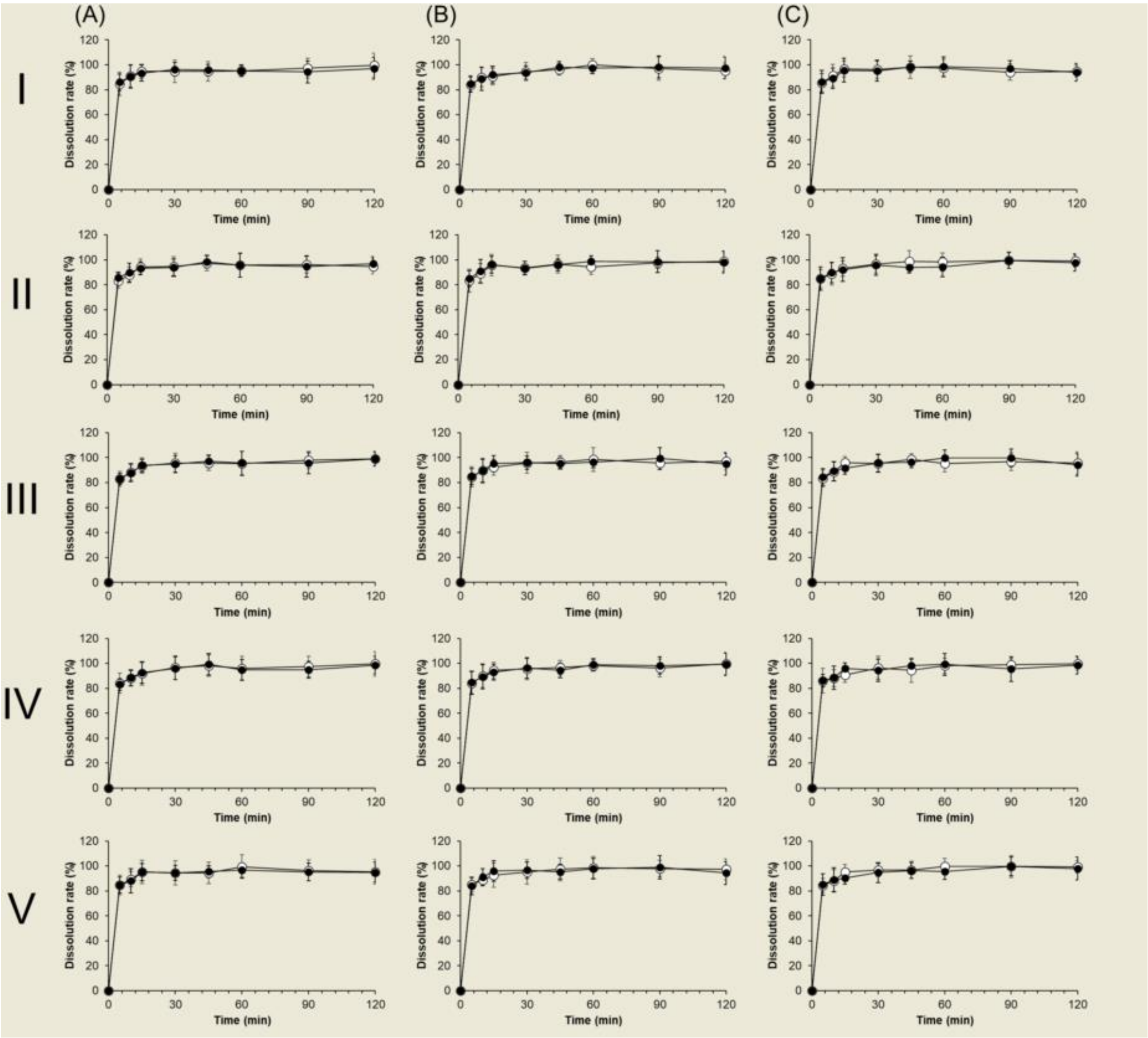

Figure S3. Dissolution profiles of compounded hydrocortisone powder in water stored in three conditions: closed bottle (A), in-use bottle (B), and laminated paper (C). Open circles indicate the dissolution rate on day 0; closed circles indicate the dissolution rate on day 120.
